# Classical Angiogenic Signaling Pathways and Novel Anti-Angiogenic Strategies for Colorectal Cancer

**DOI:** 10.3390/cimb44100305

**Published:** 2022-09-26

**Authors:** Mengyuan Cao, Yunmeng Wang, Guige Lu, Haoran Qi, Peiyu Li, Xiaoshuo Dai, Jing Lu

**Affiliations:** 1 Department of Pathophysiology, School of Basic Medical Sciences, Zhengzhou University, Zhengzhou 450001, China; 2 Collaborative Innovation Center of Henan Province for Cancer Chemoprevention, Zhengzhou University, Zhengzhou 450001, China; 3 State Key Laboratory of Esophageal Cancer Prevention & Treatment, Zhengzhou University, Zhengzhou 450052, China

**Keywords:** colorectal cancer, angiogenesis, VEGF, NF-κB, JAK-STAT, Wnt, Notch

## Abstract

Although productive progress has been made in colorectal cancer (CRC) researchs, CRC is the second most frequent type of malignancy and the major cause of cancer-related death among gastrointestinal cancers. As angiogenesis constitutes an important point in the control of CRC progression and metastasis, understanding the key signaling pathways that regulate CRC angiogenesis is critical in elucidating ways to inhibit CRC. Herein, we comprehensively summarized the angiogenesis-related pathways of CRC, including vascular endothelial growth factor (VEGF), nuclear factor-kappa B (NF-κB), Janus kinase (JAK)/signal transducer and activator of transcription (STAT), Wingless and int-1 (Wnt), and Notch signaling pathways. We divided the factors influencing the specific pathway into promoters and inhibitors. Among these, some drugs or natural compounds that have antiangiogenic effects were emphasized. Furthermore, the interactions of these pathways in angiogenesis were discussed. The current review provides a comprehensive overview of the key signaling pathways that are involved in the angiogenesis of CRC and contributes to the new anti-angiogenic strategies for CRC.

## 1. Introduction

Colorectal cancer is the second most frequent type of malignancy and the major cause of cancer-related death among gastrointestinal cancers, despite significant advances over the past two decades in preventive screening and therapy aimed at improving patient survival [1]. The overall survival rate in patients is still low, particularly the patients who are diagnosed at an advanced stage. In terms of cancer etiology and CRC, the mechanism of cancer development in most patients remains unclear. Many signaling pathways in CRC are implicated in the regulation of several biological processes, including cell proliferation, differentiation, angiogenesis, apoptosis, and survival [2].

Judah Folkman’s early observations showed that fast-growing tumors have a large number of blood vessels compared to dormant tumors, leading to the concept that tumor progression requires the initiation of tumor angiogenesis and anti-angiogenesis [3]. Through further studies, Folkman isolated an inducer of tumor angiogenesis and speculated that inhibition of the angiogenesis might block the formation of new blood vessels and arrest solid tumors at a very small size [4]. This point of view set off a wave of research on tumor angiogenesis.

As a hallmark of cancer, angiogenesis is closely related to tumor growth, metastasis, invasion, prognosis, and recurrence [5,6,7,8]. Tumor cells need enough oxygen and nutrients to survive, and angiogenesis can provide them with these substances to promote cell growth and proliferation [9]. To promote angiogenesis, cancer cells often overexpress angiogenic factors, such as VEGFA [10]. However, in the process of tumor occurrence, the abnormalities and disorders of vessels structure and function often occur, which not only promote the metastasis of tumor cells but also hinder the delivery of chemotherapy drugs to tumor tissues, leading to the occurrence of chemotherapy drug resistance [11].

Angiogenesis is a crucial process in CRC development, in which VEGF signaling pathway is a classical regulatory pathway in CRC [12]. Thus, in previous reviews, treatment has mainly been targeted on the regulators of this pathway. In fact, many studies suggest that multiple pathways including VEGF signaling pathway have targets of high therapeutic value to regulate CRC angiogenesis. However, the mechanism of angiogenesis in CRC is not very clear at present. Therefore, understanding the mechanism of angiogenesis in CRC is of great significance for inhibiting the occurrence and development of tumors, resolving the resistance to various drugs, and even developing new antiangiogenic drugs. This review focuses on the role of the VEGF, NF-κB, JAK-STAT, Wnt, and Notch signaling pathways in angiogenesis in CRC.

## 2. VEGF Signaling Pathway in CRC

### 2.1. Introduction to the VEGF Signaling Pathway

The VEGF signaling pathway is established as one of the key regulators of tumor angiogenesis [13]. The VEGF/VEGF receptor axis is composed of multiple ligands and receptors, including ligands VEGFA, VEGFB, VEGFC, VEGFD, and placental growth factor (PLGF) and receptors VEGFR-1, VEGFR-2, and VEGFR-3 [13,14,15]. VEGFA is the predominant proangiogenic factor in CRC and is associated with metastases formation and poor prognosis in CRC patients [16]. More specifically, VEGF can regulate the formation of blood vessels and participate in the physiological activities of endothelial cells by binding to VEGFR to activate downstream signals such as PI3K/AKT and mitogen-activated protein kinase (MAPK) [17,18,19].

### 2.2. Factors Promoting CRC Angiogenesis by the VEGF Signaling Pathway

**Figure 1:**
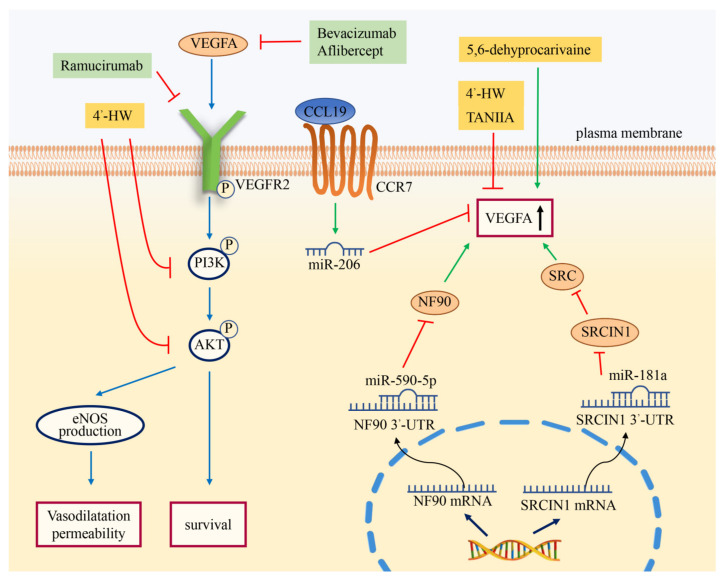
Regulation of angiogenesis via the VEGF signaling pathway in CRC. As monoclonal antibodies to VEGFA or VEGFR2, bevacizumab, aflibercept, and ramucirumab can inhibit angiogenesis in CRC by binding to their corresponding molecules. The chemokine CCL19 can promote miR-206 to inhibit VEGFA in a CCR7-dependent manner. MiR-181a and miR-590-5p inhibit the expression of related target molecules by binding to the target 3’-UTR. These processes can affect the expression of VEGFA. 4’-HW can block PI3K and AKT phosphorylation and inhibit VEGFA expression. The compounds Tan IIA and 5,6-dehyprocarivaine can also affect the increase in VEGFA.

Many biologically active molecules promote angiogenesis by regulating the VEGF signaling pathway. It has been well established that the HIF-1 was a key mediator of hypoxic responses. Under hypoxia, HIF-1 could significantly upregulate the expression of VEGF [20,21]. As the core subunit of SWI/SNF (switch/sucrose nonfermentable) family complexes, brahma-related gene 1 (BRG1) was significantly overexpressed in CRC tissues compared with normal tissues. The study showed that BRG1 could promote VEGFA expression by interacting with HIF-1α to increase CRC angiogenesis [22]. As a key member of the Gab family, Grb2-associated binder 2 (Gab2) is a scaffolding protein that serves as a critical signaling amplifier downstream of tyrosine kinase receptors [23,24]. A study demonstrated that Gab2 expression was positively correlated with the levels of VEGF in CRC tissues. Gab2 could upregulate the expression of VEGF and activate the MEK/ERK/c-Myc pathway, thus promoting angiogenesis in CRC [25]. As a non-receptor protein tyrosine kinase, non-receptor protein tyrosine kinase (SRC) was activated following engagement of many different classes of cellular receptors, including receptor protein tyrosine kinases, G protein-coupled receptors, as well as cytokine receptors. A large amount of evidence indicated that SRC regulated angiogenesis via the SRC-STAT3-VEGF pathway in CRC [26]. It has been demonstrated that SRC could induce the expression of HIF-1α and then upregulate the expression of VEGFA [27]. In addition, SRC could stabilize the content of HIF-1α under normoxic conditions by inhibiting prolyl hydroxylation [28]. Previous studies have shown that miR-181a inhibited SRC kinase signaling inhibitor 1 (SRCIN1) to increase the activity of SRC (Figure 1) [26]. This process promoted the secretion of VEGF, which in turn induced angiogenesis in CRC. Serum response factor (SRF), a member of the MADS box superfamily of transcription factors, could promote tumor metastasis by enhancing the proliferation, invasion, and angiogenesis of tumor cells. As a key condition of VEGF-induced angiogenesis, SRF could act as an upstream regulator to affect the expression of VEGF. SRSF3 could bind to SRF pre-mRNA and participate in the splicing of SRF pre-mRNA, thereby promoting angiogenesis of CRC through this process [29]. CircRNAs can regulate various functions and gene expressions through sponging with microRNAs (miRNAs). Circ_0056618, a novel circRNA discovered in gastric cancer, has been reported to promote angiogenesis in CRC [30]. MiR-206 is a miRNA that can regulate invasion, proliferation, and angiogenesis by binding to the 3′ untranslated regions (UTR) of Met to inhibit Met/ERK/Elk-1/HIF-1α/VEGFA signaling in CRC [30,31]. Consistently, Zheng et al. demonstrated that circ_0056618 promoted the expression of VEGFA through sponging with miR-206 in CRC. Besides, it has also been shown that 5,6-dehydrocarvaine extracted from the rhizome of Galangal could induce the expression of VEGF in HT-29 cells, thereby promoting cancer angiogenesis [32]. As a high-production-volume industrial chemical, Bisphenol A (BPA) is widely distributed in air, soil, water, and sewage sludge. It has been reported that BPA exposure promoted the excessive production of reactive oxygen species (ROS), which in turn activated the HIF-1α/VEGF/PI3K/ AKT axis in CRC cells [33].

### 2.3. Factors Inhibiting CRC Angiogenesis by the VEGF Signaling Pathway

In addition, some molecules that inhibit angiogenesis through the VEGF signaling pathway have been described. These include chemokine CC ligand 19 (CCL19), also known as macrophage inflammatory protein 3-β (MIP-3B). The expression of CCL19 in CRC tissues was lower than that in normal tissues, and negatively correlated with cancer angiogenesis. CCL19 inhibited the Met/ERK/ELK-1/HIF-1α/VEGFA pathway by promoting the expression of miR-206 (Figure 1). CCL19 inhibited angiogenesis in CRC through this process [31]. Some microRNAs can also inhibit angiogenesis in CRC through the VEGF pathway. It was found that the expression of miR-590-5p was significantly downregulated in CRC compared with normal tissues. The direct target of miR-590-5p is nuclear factor 90 (NF90), which is a protein synthesis regulator of VEGF (Figure 1). MiR-590-5p could inhibit CRC angiogenesis and metastasis by inhibiting the NF90/VEGFA axis [34]. Besides, miR-148a has been shown to downregulate VEGF via the pERK/HIF-1α pathway [35].

### 2.4. Anti-Angiogenic Therapy of the VEGF Signaling Pathway

In CRC treatment, antiangiogenic therapy is an important method, and the VEGF signaling pathway plays a significant role. Bevacizumab is an FDA-approved antiangiogenic agent targeting VEGF for the treatment of metastatic colorectal cancer (MCRC) (Figure 1) (Table 1) [36]. Studies have shown that the overall response rate and disease control rate of bevacizumab combination therapy in 35 CRC patients were 3.2% and 51.6%, respectively [37]. Aflibercept is also an antiangiogenic drug already in use. Unlike bevacizumab, it can target not only VEGFA but also VEGFB and PLGF [38]. Ramuciumab is a monoclonal antagonist targeting VEGFR2. It inhibits angiogenesis by blocking VEGFA binding to VEGFR2 [39]. By inhibiting angiogenesis, other molecules can also play therapeutic roles. A compound called cantharidin (CTD) secreted by Blister Beetle inhibited angiogenesis by inhibiting the phosphorylation of JAK1, ERK, and AKT and the activation of STAT3 signaling induced by VEGF [40]. Scopoletin, the main bioactive component of the *Erycibe obtusifolia* Benth, inhibited angiogenesis by blocking the autophosphorylation and downstream signaling pathways of VEGFR2 [41]. In athymic nude mice bearing HCT-116 cells, scopoletin had a significant inhibitory effect on tumor vasculature. Tumors collected from nude mice showed a dramatic decrease in blood vessel density compared to the control group [42]. Toll-like receptor 4 (TLR4) could induce the PI3K/AKT signaling and play a central role in the progression of CRC [43]. Moreover, baicalein directly bound to TLR4 to inhibit TLR4/HIF-1α/VEGF signaling pathway and angiogenesis in CRC [44]. Ginkgetin and resveratrol could inhibit VEGF-mediated angiogenesis during tumorigenesis (Figure 1). These two molecules inhibited the phosphorylation of VEGFR2, AKT, endothelial nitric oxide synthases (eNOS), and ERK in human umbilical vein endothelial cells (HUVECs) [45]. Tanshinone IIA (Tan IIA) could reduce the expression of angiogenin, VEGF and basic fibroblast growth factor (bFGF) in CRC to inhibit angiogenesis [46]. The study showed that 4’-hydroxywogonin (4’-HW) could not only decrease the mRNA and protein expression of VEGFA but also inhibit the phosphorylation of PI3K and AKT in CRC cells. This suggested that 4’-HW was a promising anticancer drug targeting angiogenesis in CRC [47].

## 3. NF-κB Signaling Pathway in CRC

### 3.1. Introduction to the NF-κB Signaling Pathway

NF-κB subunits in mammals are divided into five molecules: p65/RelA, RelB, c-Rel/Rel, NF-κB1 (p50 and its precursor p105), and NF-κB2 (p52 and its precursor p100) [64,65]. The NF-κB pathway has two distinct but interacting branches: the canonical pathway, which is activated by tumor necrosis factor-α (TNF-α), Toll-like receptor ligands, interleukin-1 (IL-1) and angiotensin II, and the non-canonical pathway, which is activated by the TNF superfamily members B cell-activating factor (BAFF), CD40, receptor-activated NF-κB ligand (RANKL), lymphotoxin β, and RNA viruses [49,66]. The transcriptional activity of NF-κB requires the formation of homo- or heterodimeric protein complexes [64], such as p65:p50 heterodimers and p52:RelB heterodimers; the former acts on the canonical pathway, while the latter participates in the non-canonical pathway [67].

In general, the inhibitor IκB in the cytoplasm of most resting cells binds to NF-κB, covers the nuclear localization sequence (NLS) of NF-κB, blocks DNA binding and nuclear localization, and leads to its inactivation in the cytoplasm. When stimulated by extracellular stimuli such as bacteria, viruses, and cytokines, the IκB kinase (IKK) complex upregulates and phosphorylates IκB, contributing to the degradation of IκB in the proteasome [66,67,68]. NF-κB is subsequently exposed to the NLS, enters the nucleus and binds to a specific DNA sequence, triggering downstream gene expression [66]. However, for the non-canonical NF-κB signaling pathway, the key to its activation lies in the transformation of p100 to p52 and the formation of heterodimers with RelB, which depends on NF-κB-inducing kinase (NIK) and IKKα-mediated phosphorylation of p100 [65,67,68].

Activation of the NF-κB signaling pathway is closely associated with the progression of CRC, such as cell proliferation, apoptosis, angiogenesis, and metastasis. Therefore, NF-κB is usually considered a therapeutic target.

### 3.2. Factors Promoting CRC Angiogenesis by the NF-κB Signaling Pathway

**Figure 2:**
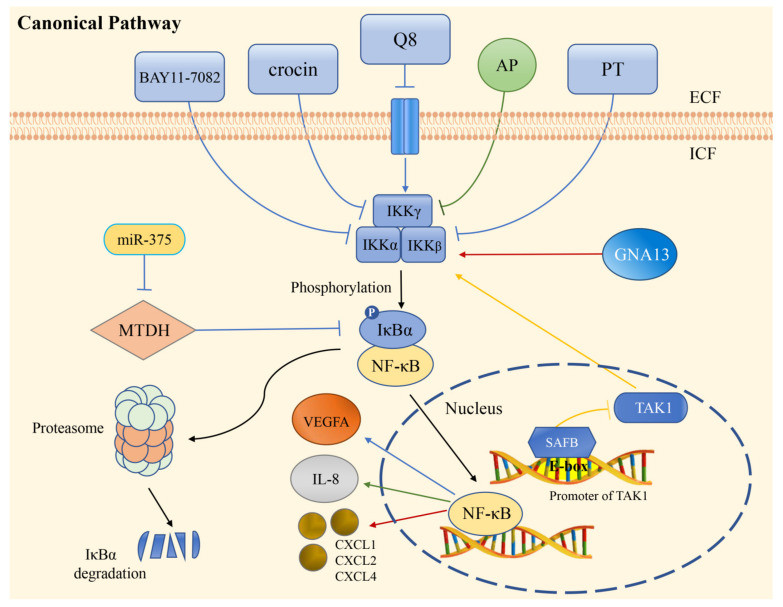
Regulation of angiogenesis via the canonical NF-κB pathway in CRC. The extracellular compounds BAY11-7082, crocin, Q8, and PT inhibit the expression of VEGFA by suppressing the phosphorylation of IκB. While GNA13 promotes angiogenesis through the regulation of chemokines production. AP restrains IL-8 production and angiogenesis by inhibiting the degradation of IκB, then prevents NF-κB from entering the nucleus. SAFB targets the promoter of TAK1 in the nucleus and inhibits angiogenesis. The expression of miR-375 can downregulate MTDH level, leading to the inhibitory effect of IκBα on NF-κB. Q8: IUPAC name (E)-2-(2-quinolin-2-yl-vinyl)-benzene-1,4-diol HCl; AP: Andrographolide; PT: Parthenolide; IKK: IκB kinase complex; SAFB: scaffold attachment factor B; MTDH: metadherin.

Angiogenesis plays an important role in the migration and invasion of solid tumors. The NF-κB pathway regulates the expression of many angiogenesis regulatory factors, such as VEGF, PDGF-BB, MMP-2, MMP-9, CXCL1, CXCL8, IL-8, and COX-2, ultimately regulating tumor angiogenesis [9,49]. A recent study validated that the overexpression of B7-H3 increased the activity of NF-κB through a luciferase reporter assay and found that the expression of VEGFA in B7-H3-induced CRC cells was regulated by the NF-κB pathway [9]. In summary, B7-H3 induced VEGFA expression by activating the NF-κB pathway, which ultimately promoted CRC angiogenesis. CXCL5 belongs to the CXC-type chemokine family, and studies showed that CXCL5 regulated the expression of FOXD1 and VEGFA and promoted tumor angiogenesis by activating the AKT/NF-κB pathway [69]. In addition, activation of the AKT/NF-κB pathway might be involved in CCR6-mediated tumor angiogenesis, thereby promoting the secretion of VEGFA [70]. Previous studies reported that protein phosphatase of regenerating liver-3 (PRL-3) might be associated with triggering angiogenesis and establishing the microvascular system [71]. In addition, PRL-3 in cancer cells could upregulate the secretion of IL-6 and IL-8 through MAPK signals acting on tumor-associated macrophages. Finally, the NF-κB pathway was activated to promote angiogenesis in CRC cells [72]. Overexpression of GNA13 has also been shown to upregulate the expression of the chemokines CXCL1, CXCL2, and CXCL4 by activating NF-κB/p65, thus playing an important role in promoting angiogenesis in CRC (Figure 2) [73].

### 3.3. Factors Inhibiting CRC Angiogenesis by the NF-κB Signaling Pathway

It is common knowledge that members of the IκB family are closely related to the activity of NF-κB as inhibitory factors. As prototypical IκB proteins, IκBα and IκBβ could block the NF-κB pathway-mediated angiogenesis in CRC [51,52,74]. Kinases in the NF-κB signaling pathway, such as IKK and NIK, are essential for NF-κB to promote angiogenesis. Therefore, when the activity of these kinases is inhibited, the above process will be restrained. A study investigated that the specific inhibition of NF-κB activity by IKK1/2 siRNA could reduce the expression of *c-Myc* and further inhibit angiogenesis [75]. Similarly, NIK-targeting siRNA could inhibit NIK mediated non-canonical NF-κB pathway and play the role of inhibiting angiogenesis [76]. Besides, the NEMO-binding domain peptide, which is an amino-terminal α-helical region of NEMO, could block the association of NEMO (also known as IKKγ) with IKKβ and inhibit the activity of NF-κB involved in angiogenesis [77].

There are several factors that have effects against NF-κB-mediated angiogenesis in different ways. Scaffold attachment factor B (SAFB) is a transcriptional suppressor in CRC progression. It has been demonstrated in vitro and in vivo that SAFB can inhibit TAK1 activity by targeting the first E-box of its promoter, leading to the inhibition of the NF-κB signaling pathway involved in CRC invasion, metastasis, and angiogenesis (Figure 2) [74]. As a microRNA overexpressed in CRC, miR-375 could directly target metadherin (MTDH), upregulate IκBα expression, downregulate p65:p50 heterodimers levels, and thereby served to suppress angiogenesis [78].

In addition, some inhibitors of NF-κB have a similar effect of suppressing angiogenesis in CRC. PDTC could inhibit CXCL5-dependent induction of FOXD1 and VEGFA expression [69]. BAY11-7082 could significantly reduce p-IKKα and p-p65 levels, thereby inhibiting VEGFA expression [9,72]. SN50 served to downregulate the expression of MMP7 and suppress angiogenesis in CRC by blocking the NF-κB pathway [79].

### 3.4. Anti-Angiogenic Therapy of the NF-κB Signaling Pathway

It has been reported that imatinib could activate different intracellular signaling pathways, thereby breaking the feedback loop between proinflammatory cytokines and transcription factors (NF-κB, JAK3/STAT3). Imatinib also appeared to inhibit the coordination of proinflammatory cytokines by intracellular signaling, which was also involved in the upregulation of angiogenic factors in CRC (Table 1) [48]. As a natural dietary product, curcumin can be used as a chemosensitizer to inhibit angiogenesis in most cancer cells and is a promising approach for the treatment of CRC [80]. A study showed that curcumin inhibited tumors by influencing angiogenesis regulators and other molecular bases through NF-κB and then served as an antiangiogenic therapeutic pathway [50].

Prevention of angiogenesis by NF-κB-specific inhibitors has been the core of anticancer therapy. Andrographolide (AP), a natural phytochemical found in Andrographis paniculata, antagonized IL-8 induced by TNF-α by inhibiting NADPH oxidase/ROS/NF-κB and other signaling pathways, which led to the inhibition of angiogenesis in the tumor microenvironment (Table 1) [51]. Parthenolide (PT) was also found to be a NF-κB inhibitor that significantly inhibited hypoxia-dependent HIF-1α activity and angiogenesis by inhibiting NF-κB activation (Figure 2) [52]. In addition, piperine, a natural alkaloidal pungent product presented in pepper plants, has been demonstrated to modify enzymes and transcription factors activity to inhibit angiogenesis, invasion, and metastasis [81]. It could suppress the expression of IL-8 stimulated by lithocholic acid through inhibiting the transcriptional activity of NF-κB, then affecting the activity of CRC angiogenesis [82].

## 4. JAK-STAT Signaling Pathway in CRC

### 4.1. Introduction to the JAK-STAT Signaling Pathway

The JAK/STAT signaling pathway plays a critical role in various aspects of CRC, especially angiogenesis in CRC. This pathway consists of tyrosine kinase-associated receptors, JAK and STAT, which are coupled to activate. By binding with tyrosine kinase-associated receptors, over 40 different cytokines or growth factors can induce the STAT3 signaling pathway. The JAK family proteins include four members, JAK1, JAK2, JAK3, and TYK2, related to the cytoplasmic regions of tyrosine kinase-associated receptors [83]. STAT family proteins have seven members: STAT1, STAT2, STAT3, STAT4, STAT5, STAT6, and STAT7. STAT family proteins have dual functions of signal transduction and transcriptional activation [84].

The end of the active STAT signaling pathway enables a succession of gene expression changes, which then participate in biological processes, including proliferation, angiogenesis, and metastasis [85,86,87,88]. When cytokines (IL-6, IL-11, IFN, etc.) bind to their corresponding ligands, the receptors and JAK are aggregated, and the adjacent JAK is activated by mutual phosphorylation. After JAK is activated, STAT1/3/5 are activated through phosphorylation [89]. Then, STAT1/3/5 expose the NLS, enter the nucleus and in turn activate STAT1/3/5-mediated transcription of genes. When this pathway is upregulated, it can lead to angiogenesis in CRC [90,91]. It has been reported that activation of the IL-6/STAT3 pathway downregulates the expression of genes to promote tumor angiogenesis. A study suggested that the blockade of proangiogenic signaling significantly reduced colorectal tumor growth in mice with constitutive STAT3 activation in COLVI+ fibroblasts [92]. Activation of STAT3 in tumor-associated fibroblasts promotes angiogenesis in CRC. In addition, STAT2 activates the oncogenic STAT3 signaling pathway to promote CRC [93].

### 4.2. Factors Promoting CRC Angiogenesis by the JAK/STAT Signaling Pathway

**Figure 3:**
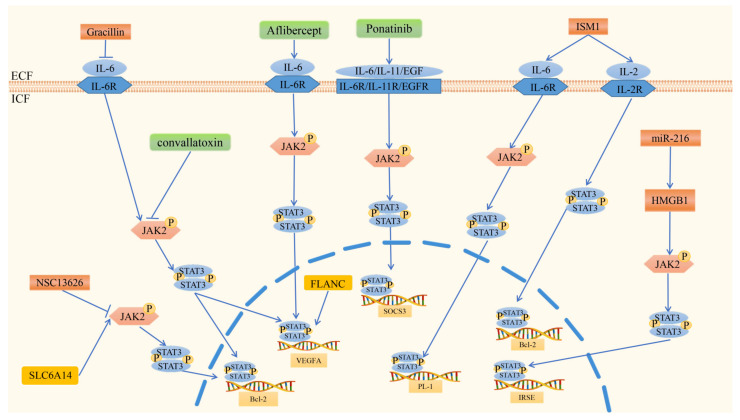
Regulation of angiogenesis via the JAK2/STAT3 signaling pathway in CRC. In the JAK2/STAT3 signaling pathway, SLC6A14 and FLANC promote angiogenesis. SLC6A14 activates JAK2 through phosphorylation, after which STAT3 is activated by mutual phosphorylation, and STAT3 then enters the nucleus and activates the transcription of Bcl-2. FLANC enters the nucleus directly and activates the transcription of VEGFA. Factors inhibiting CRC angiogenesis via JAK2/STAT3 signaling include ISM1, NSC13626, Gracillin, and miR-216. ISM1 activates the transcription of Bcl-2 via the IL2/STAT5 signaling pathway and activates PL-1 via the IL6/JAK2/STAT3 signaling pathway. NSC13626 has a negative feedback mechanism with JAK2, and the downstream target of JAK2 is p-STAT3. Gracillin inhibits the transcription of Bcl-2 and VEGFA via the IL6/JAK2/STAT3 signaling pathway. MiR-216 inhibits angiogenesis by targeting HMGB1, which mediates the JAK2/STAT3 pathway. Aflibercept, convallatoxin, and ponatinib are antiangiogenic drugs in CRC. Aflibercept modulates inflammation-related angiogenesis via the IL-6/JAK2/STAT3 axis, ultimately regulating the transcription of VEGFA. CNT also shows efficacy in the inhibition of JAK2/STAT3 to inhibit the expression of Bcl-2 and VEGFA. Ponatinib inhibits JAK2/STAT3 activity driven by EGF/EGFR, IL-6/IL-6R, and IL-11/IL-11R, regulating the transcription of SOCS3.

Promoting factors in the JAK/STAT signaling pathway play a very important role in the regulation of CRC angiogenesis. These biologically active molecules include cytokines (chemokines), various proteins, and lncRNAs (Figure 3). The agonists IL-6/IL-6R, EGF/EGFR and IGF/IGFR are three major ligand/receptor systems that drive the JAK/STAT pathway in CRC [94,95,96]. It has been reported that there is increased production of IL-6 in tumor tissues and in the serum of patients with CRC. Several studies have shown that IL-11, which has similar cellular mechanism as IL-6, is also a central regulator of STAT3 activation and angiogenesis [92].

Solute carrier family 6 member 14 (SLC6A14) expression, which is low in normal human cells, is subject to the change of the JAK2/STAT3 pathway in CRC [97,98]. SLC6A14 is overexpressed in CRC cells. The JAK2/STAT3 signaling pathway is substantially activated when SLC6A14 is overexpressed [98]. When SLC6A14 expression was blocked in vivo, researchers found that it protected against intestinal colitis-associated tumorigenesis, meaning that metastasis and angiogenesis of cancer were inhibited. Through further research, they concluded that SLC6A14 produces a manifest effect by the activation of the JAK2/STAT3 signaling pathway. In turn, it is feasible to inhibit JAK2/STAT3 signaling and reduce angiogenesis mediated by SLC6A14.

In terms of the lncRNA FLANC, there are connections with the JAK/STAT3 signaling pathway. FLANC is a primate-specific lncRNA residing within the first intron of Cadherin EGF LAG seven-pass G-type receptors 1 (CELSR1), which is weakly expressed in normal colon cells [99]. This gene encodes a protein that is one of the components of the cadherin superfamily [100]. In CRC cells, FLANC expression was much higher than that in normal colon cells [101]. Of note, FLANC is a significant promoting factor of angiogenesis in vitro and in vivo. Mechanistically, FLANC overexpression prolongs the half-life of phosphorylated STAT3 (pSTAT3 at Tyr705) and induces VEGFA transcription, which is a key regulator of angiogenesis [102,103,104].

### 4.3. Factors Inhibiting CRC Angiogenesis by the JAK/STAT Signaling Pathway

Various factors inhibiting angiogenesis via the JAK/STAT3 signaling pathway in CRC have been described. These include proteins, plant-based compounds, and RNAs (Figure 3).

Na^+^/H^+^ exchanger regulatory factor 2 (NHERF2) is an angiogenesis-inhibiting protein. The NHERF family of proteins are scaffolds that orchestrate the interaction of receptors and cellular proteins [105]. A study found that NHERF2 expression was increased in advanced-stage CRC to upregulate the phosphorylation of STAT3 through the IL-6-JAK-STAT3 pathway [106]. Notably, the absence of NHERF2 in vivo decreases STAT3 activation and tumor growth, which means that NHERF2 may be a potential target for cancer treatment. A relationship between the upregulation of Isthmin 1 (ISM1) in CRC and tumor angiogenesis was also described. A study by Yuhui Wu on the effect of ISM1 in CRC showed that ISM1 was highly associated with immune-related pathways, such as the IL-2/STAT5 and IL-6/JAK/STAT3 signaling pathways [107]. They also demonstrated that angiogenesis was significantly positively associated with ISM1 [108]. It was proven that compound NSC13626 inhibited CRC cell growth through a negative feedback mechanism with JAK and arrested the cell cycle in the S phase [109,110]. The downstream targets of JAK2 include STAT3 and STAT5, in which STAT3 signaling plays an important role in angiogenesis of CRC [111].

Certain plant-based compounds with an inhibitory influence on angiogenesis have also been described. Genistein, a chemopreventive phytochemical drug against CRC, has efficient interactions with STAT proteins. In vitro, significant suppression of cell proliferation and STAT3 protein expression has been shown after treatment with genistein [112]. The curcumin derivative 5Br-6b can inhibit the proliferation of CRC cells by blocking the activation of STAT3 and its target gene [113]. Gracillin exerts potent anticancer and antiangiogenic effects against CRC by inhibiting the IL-6/STAT3 pathway [114]. A study found that curcumin combined with (−)-epigallocatechin-3-gallate (EGCG) reduced tumor growth and angiogenesis by inhibiting the JAK/STAT3/IL-8 signaling pathway in CRC [115]. Noncoding RNAs are also responsible for modulating protein-coding gene expression related to this signaling. Upregulation of lncRNA RP11-468E2.5 interacts with STAT5 and STAT6 and inhibits the JAK/STAT signaling pathway to affect the progression of CRC [116]. MiR-216 is another tumor suppressor that inhibits angiogenesis by targeting high mobility group box 1 (HMGB1). In turn, HMGB1 is strongly expressed in CRC and mediates the JAK2/STAT3 pathway [117].

### 4.4. Anti-Angiogenic Therapy of the JAK/STAT Signaling Pathway

As previously stated, the STAT signaling molecule enters the nucleus to modulate the transcription of target genes, especially VEGFA. JAK/STAT signaling also offers potential sites for antiangiogenic therapy, such as JAK1, JAK2 [56], STAT1 [118], STAT3 [119], and STAT5. There are several antiangiogenic drugs that can act against these therapeutic targets. Aflibercept (Figure 3) (Table 1) modulates inflammation-related angiogenesis via the IL-6-STAT3 axis. A recent study showed that IL-6 expression generates a position feedback loop with VEGF [53]. Therefore, neutralization of VEGF with aflibercept decreases the activation of STAT3 and reduces IL-6 expression levels 24 h after treatment of HUVECs with the drug [120]. CNT also shows efficacy in the inhibition of the JAK2/STAT3 (T705) and mTOR/STAT3 (S727) signaling pathways in CRC [56]. Interestingly, the current results reveal that crosstalk between the two signaling pathways can collaboratively regulate STAT3 activation and that CNT plays a role in this process. Ponatinib, as a lead candidate, inhibits STAT3 activity driven by EGF/EGFR, IL-6/IL-6R, and IL-11/IL-11R. Likewise, ponatinib inhibits CRC migration and tumor growth compared with control-treated mice [54]. Chemotherapy-based comprehensive treatment is the usual way to treat CRC [121]. Napabucasin is a chemoradio-sensitizer for CRC, but it inhibits angiogenesis through an ROS-mediated effect and alteration of STAT3 signaling [55].

## 5. Wnt Signaling Pathway in CRC

### 5.1. Introduction to the Wnt Signaling Pathway

The Wnt family is a group of proteins that act in many cellular functions including organ formation, stem cell renewal, and cell survival [122]. The gain or loss of function of the Wnt signaling pathway can result in angiogenesis and abnormal vascular development [123].

The Wnt signaling pathway is classified as canonical pathway and non-canonical pathways. In the canonical pathway, Wnt signaling is activated by binding to Wnt proteins to surface receptors composed of the seven transmembrane frizzled proteins and the low-density lipoprotein receptor-related protein 5/6 (LRP5/6). After binding, the cytoplasmic protein disheveled (Dvl) is activated. The activation of Dvl induces the dissociation of glycogen synthase kinase 3β (GSK-3β) from Axin and causes the inhibition of GSK-3β. In the Wnt signaling pathway, the level of β-catenin was controlled by the “destruction complex” composed of Axin, GSK3β, casein kinase 1α (CK1α), APC, etc. [124]. Because of the inactivation of the “destruction complex”, the phosphorylation and degradation of β-catenin was inhibited. Then, stabilized β-catenin was translocated into the nucleus and led to the transcription of target genes such as *c-Myc* and cyclin D1 [125]. Furthermore, the canonical Wnt signaling pathway is correlated with angiogenesis and vascular differentiation, which is important in vascular sprouting and network maturation.

The major non-canonical pathways contain Wnt/Ca^2+^ and Wnt/PCP pathways. In the Wnt/Ca^2+^ pathway, Wnt binds to Frizzled and activates Dvl, causes the release of Ca^2+^ from the endoplasmic reticulum, activates Ca^2+^ binding proteins including PKC and CamKII. Signal transduction activates the nuclear factor of activated T cells (NFAT) through Ca^2+^. The Wnt/PCP pathway is mediated by the GTPases RhoA and Ras and can exert effects on the cytoskeleton through the ROCK axis.

### 5.2. Factors Promoting CRC Angiogenesis by the Wnt Signaling Pathway

Many factors have been shown to promote CRC angiogenesis through the Wnt signaling pathway. Transglutaminase 2 (TGM2) is a novel molecular marker that is important for the therapy and prognosis of CRC. In CRC, the expression of TGM2 was higher than that in normal tissues [126]. TGM2 could promote angiogenesis and upregulate the expression of Wnt3a, β-catenin, and cyclin D1. When TGM2 was inhibited, the apoptosis of CRC cells was promoted and then inhibited the angiogenesis of cancer [127]. Transmembrane-4 L-six family member-1 (TM4SF1), the founding member of the TM4SF, is an antigen regulated by oncogenes [128]. TM4SF1 expression was higher in CRC tissues than in non-tumor tissues and was positively correlated with poor prognosis. TM4SF1 regulated SOX2 via the Wnt/β-catenin/c-Myc/SOX2 signaling pathway [129]. In addition, with the knockdown of TM4SF1, the expression of *c-Myc* and epithelial to mesenchymal transition (EMT) were suppressed [129].

Tumor associated macrophages (TAMs) had a leading position in the tumor microenvironment (TME), which was closely correlated with tumor initiation, progression, and metastasis [130]. TAMs could release plenty of cytokines, including IL-1β, CXCL-4, CXCL-8, and CXCL-12. These cytokines synergistically regulated endothelial cells, matrix remodeling, and vascularization in CRC angiogenesis [130]. IL-1β could inactivate GSK3β by inducing the phosphorylation of AKT and PDK1. This process enhanced TCF4/β-catenin transcriptional activity and activated Wnt target genes in CRC cells, such as *c-Myc* and *c-Jun* [131].

**Figure 4:**
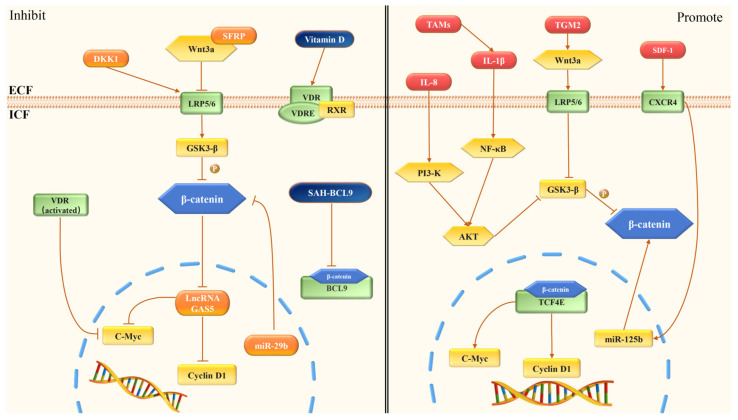
Regulation of angiogenesis via the Wnt/β-catenin signaling pathway in CRC. DKK1 acts on LRP5/6. LRP5/6 causes the degradation of β-catenin. SFRPs combine with Wnt3a to inhibit the expression of β-catenin. LncRNA GAS5 reduces the expression of β-catenin, *c-Myc*, and cyclin D1. MiR-29b downregulates the expression of β-catenin. Vitamin D can activate VDR, and the activated VDR inhibits the expression of *c-Myc*. SAH-BCL9 dissociates native β-catenin/BCL9 complex and then suppresses Wnt transcription selectively. IL-8 acts on AKT/GSK3β/β-catenin, which promotes the Wnt/β-catenin pathway. TGM2 upregulates the expression of Wnt3a, β-catenin, and cyclin D1. TAMs release IL-1β, which acts on NF-κB/AKT/GSK3β and promotes Wnt signaling. SDF-1 activates CXCR4, which causes the expression of miR-125b and activates the Wnt/β-catenin signaling pathway.

It has been shown that IL-8 (also known as CXCL-8) promotes the formation of the TME, which could facilitate the generation of tumor cells and the invasiveness of cancer by enhancing the level of arginase in myeloid-derived suppressor cells. Moreover, IL-8 could promote the AKT/GSK3β/β-catenin/MMP7 pathway in CRC by upregulating BCL-2 (Figure 4) [132]. CXCL-12 could activate CXC chemokine receptor 4 (CXCR-4), which was correlated with the invasion of CRC cells. The expression of CXCR-4 in CRC cells was much higher than that in normal tissues [133]. Further studies have shown that the activation of CXCL-12/CXCR-4 axis in vascular endothelial cells could stimulate the angiogenesis through the upregulation of the Wnt/β-catenin signaling pathway [134].

### 5.3. Factors Inhibiting CRC Angiogenesis by the Wnt Signaling Pathway

Dickkopf-1 (DKK-1) is a secreted protein and an extracellular inhibitor of the Wnt signaling pathway [135]. DKK-1 bound to LRP5/6, and sequestered LRP5 away from the Frizzled/LRP6 complex, thereby inhibiting the transcription of TCF/LEF and the canonical Wnt signaling pathway (Figure 4) [135,136]. DKK-1 overexpression also downregulated the expression of VEGF and decreased the microvessel density [135]. Secreted frizzled-related proteins (SFRPs) are a series of extracellular inhibitors of the Wnt signaling pathway. SFRPs are bound to Wnt/β-catenin signaling by their cysteine-rich domains. For example, SFRP-1 bound to Wnt3a to block the Wnt signaling pathway and thereby inhibited the expression of β-catenin and *c-Myc* [137]. LncRNA GAS5 is considered to be effective in the inhibition of CRC by regulating the Wnt signaling pathway (Figure 4). Research on lncRNAs in CRC showed that oe-GAS5 expression could decrease the expression of β-catenin, *c-Myc*, and cyclin D1, which inhibited the angiogenesis of CRC [138].

Evidence suggests that the synthetic role of miR-29b was essential for the inhibition of CRC cells [139]. MiR-29b downregulated the expression of BCL9L by targeting the 3’UTR of BCL9L. BCL9L, TCF7L2, and Snail are coactivators of β-catenin. Thus, it was proposed that miR-29b inhibited the expression of many coactivators and downstream targets of CTNNB1/Wnt signaling in CRC cells. This caused the inhibition of the cell growth, tumor angiogenesis and EMT [140].

### 5.4. Anti-Angiogenic Therapy of the Wnt Signaling Pathway

Vitamin D (1,25(OH)_2_D_3_) has the potential as a therapy for CRC by inhibiting the angiogenesis through the Wnt pathway (Table 1). Vitamin D combines with vitamin D receptor (VDR), and VDR binds to retinoid X receptor (RXR). The VDR-RXR heterodimer bound to the vitamin D response element (VDRE) and participated in antineoplastic properties [57]. After being activated, VDR was associated with β-catenin and inhibited the expression of *c-Myc*, which was a downstream signal of the Wnt pathway [58]. With the lower expression of *c-Myc*, vitamin D suppressed the development of angiogenesis in CRC. In one research, for vitamin D vs. no vitamin D, the expression of β-catenin decreased by an estimated 3% (*p* = 0.41) in the full length of the colon crypts [141].

B-cell CLL/lymphoma 9 (BCL9) was regarded as a co-activator of the Wnt/β-catenin signaling pathway by participating in TCF-mediated transcription in CRC [142]. Stabilized Alpha-Helix of BCL9 (SAH-BCL9) was developed by Takada’s team in order to block the interaction with BCL9. It was reported that SAH-BCL9 had peptides from A to C and SAH-BCL9 peptide B (SAH-BCL9B) was the most effective. SAH-BCL9B could target β-catenin and dissociate native β-catenin/BCL9 complex selectively, then suppressed the transcription activity of Wnt (Table 1). By targeting the disruption of BCL9/β-catenin, it inhibited the proliferation, angiogenesis, and migration of CRC cells [60]. Due to the high binding of serum proteins, SAH-BCL9 peptides did not have pharmacokinetic properties conducive to clinical development. However, a novel β-catenin/BCL9 complex inhibitor E722-2648 is considered more effective than SAH-BCL9B [143]. In a previous study, E722-2648 could inhibit β-catenin/BCL9 complex formation and E722-2648 treatment could significantly reduce the tumor growth in the mice compared to the control group. It shows that E722-2648 may have more important implications for the development of novel therapies in CRC.

## 6. Notch Signaling Pathway in CRC

### 6.1. Introduction to the Notch Signaling Pathway

**Figure 5:**
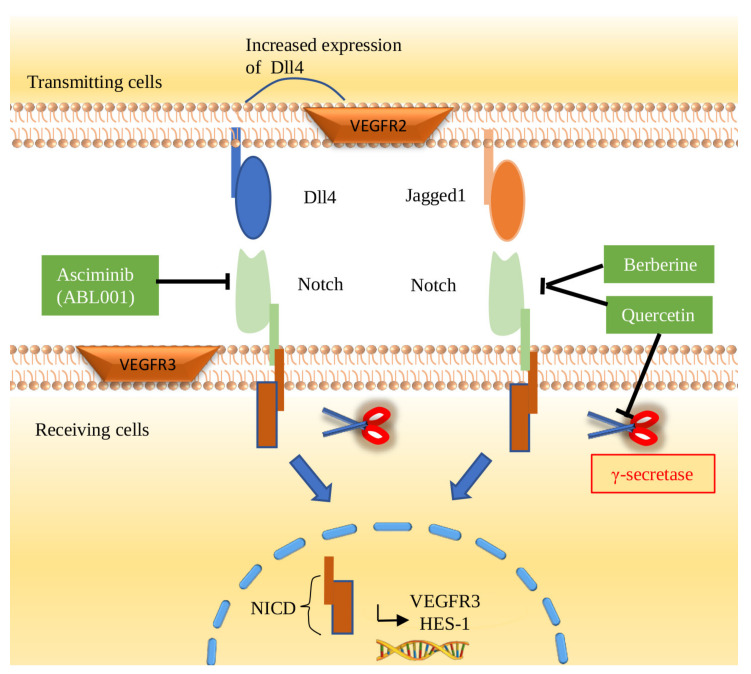
Regulation of angiogenesis via the Notch signaling pathway in CRC. Notch1 is a single transmembrane protein located on the cell surface that interacts with Dll4 and Jagged1 expressed on adjacent cells. As a result, γ-secretase is activated to split Notch to induce NICD into the nucleus. Then, VEGFR3 and HES-1 are upregulated and are involved in subsequent angiogenesis in CRC. As inhibitors of the Notch pathway, ABL001, berberine, and quercetin are considered potential therapeutic agents to inhibit angiogenesis in CRC.

The Notch signaling pathway is one of the most important signaling modes that plays a part in physiological and tumor pathology. It is not only essential for differentiation, proliferation, and apoptosis, but also angiogenesis, tip/stalk cell selection, and arteriovenous specification [144,145]. There are a series of ligands and receptors in the Notch signaling pathway. Jagged-1, Jagged-2, Delta-like-1 (Dll1), Delta-like-3 (Dll3), and Delta-Like-4 (Dll4) are ligands of the Notch signaling pathway. Meanwhile, there are four receptors in this signaling pathway: Notch-1, -2, -3, and -4. Ligand receptor binding causes γ-secretase activation. As a result, the Notch intracellular domain (NICD) enters the nucleus and binds to the related transcription factors to affect downstream target genes (Figure 5). The abnormal function of the Notch signaling pathway in CRC depends on the overexpression of ligands and receptors compared with normal tissues [146,147].

### 6.2. Factors Promoting CRC Angiogenesis in the Notch Signaling Pathway

The Notch signaling pathway is regularly altered in many cancers, as well as CRC [148]. In the endothelial cells of CRC, the Notch signaling pathway can be activated by overexpression of associated ligands and receptors, such as Dll4 and Notch1 (Figure 5). In general, activation of the Notch signaling pathway has been shown to play a role in the development and angiogenesis of CRC [149]. Once the Notch signaling pathway is activated, NICD enters the nucleus, leading to upregulation of HECS-1 and VEGFR3, which promotes subsequent angiogenesis in CRC cells [150,151]. Several factors can promote angiogenesis through the Notch signaling pathway. HDAC5 is an important factor of the histone deacetylase (HDAC) family. HDAC5 activates the Notch signaling pathway and promotes angiogenesis of CRC cells by upregulating the expression of Dll4 [152]. Currently, it has been reported that leptin can induce the expression of Notch1-4/Jagged-1/Dll4, IL-1 and VEGF/VEGFR2. In CRC, leptin promotes angiogenesis through the Notch, IL-1, and leptin crosstalk outcome (NILCO) pathway [118].

### 6.3. Factors Inhibiting CRC Angiogenesis in the Notch Signaling Pathway

It is highly important to explore the factors that inhibit angiogenesis in CRC by inhibiting the Notch signaling pathway. γ-Secretase and Dll4 are the most common targets of small-molecule inhibitors or antibodies for the Notch signaling pathway blockade [153]. An increasing number of inhibitors and monoclonal antibodies against Dll4 have been discovered. α-Mangostin has been shown to have an antiproliferative effect on cancer cells. α-Mangostin-encapsulated PLGA nanoparticles (Mang-NPs) is formulated in order to enhance the biological effect of α-Mangostin. Current studies have shown that α-Mangostin and Mang-NPs could inhibit the Notch signaling pathway by inhibiting the expression of Notch1, Notch2 and their ligand Dll4 in CRC. As a result, α-Mangostin can be used for the treatment and prevention of CRC [154,155]. Selenium binding protein 1 (SELENBP1) is frequently downregulated in tumor vessels in CRC. A study found that SELENBP1 could inhibit angiogenesis by binding with Dll4 and antagonizing the Dll4/Notch1 signaling pathway in CRC, which made SELENBP1 a potential tumor suppressor [156]. In addition, Torin-1 is an inhibitor of mammalian target of rapamycin (mTOR), which has been considered an important regulator of cancer. As markers related to angiogenesis, Notch1 and Dll4 were significantly reduced in a Torin-1-treated group compared with a control group, suggesting that Torin-1 has an anti-angiogenesis effect by inhibiting the Notch signaling pathway. Therefore, Torin-1 is considered an effective candidate drug for metastatic CRC therapy [157]. Portulaca oleracea significantly downregulates the expression of the Notch1 and β-catenin genes in CRC. The results of this study showed that Portulaca oleracea extract inhibited the growth of CRC stem cells in a dose-dependent manner by inhibiting the Notch signaling pathway, thus playing an important role in preventing angiogenesis in CRC [158]. In addition, ethanol extracted from radix of Actinidia chinensis (EERAC) was also found to suppress the expression of Notch1 and Jagged1 to inhibit angiogenesis in CRC. Meanwhile, EERAC inhibits mastermind-like transcriptional coactivator 1 (MAML1), which can activate the Notch signaling pathway [159]. In general, more molecules blocking angiogenesis of CRC by inhibiting the Notch signaling pathway are expected. 

### 6.4. Anti-Angiogenic Therapy of the Notch Signaling Pathway

Targeting the Notch signaling pathway to prevent angiogenesis in CRC is feasible (Figure 5). As a natural product, berberine (BBR) has been reported to treat diarrhea and gastroenteritis in the clinic. In recent years, BBR has been shown to have some anticancer effects [160]. For CRC, BBR inhibited the Notch signaling pathway by downregulating the expression of Notch1 in SW480 cells. Inhibition of Notch1 increased the expression of the tumor suppressor gene PTEN to inhibit CRC by regulating angiogenesis, transcription, translation, and cell cycle progression [61]. This suggested the significance of BBR as an existing drug in the treatment of CRC (Table 1) [161]. As a bispecific antibody, ABL001 has been reported to block the Dll4/Notch signaling pathway to play a superior anticancer role through an antiangiogenic effect [62]. Yana Li offered a therapeutic strategy for CRC treatment that combined quercetin (20 μM) and IR (5 Gy). Quercetin enhanced the radiosensitivity of CRC by regulating the related proteins. In CRC cells, this strategy reduced the expression of Notch1 and all five proteins of the γ-secretase complex to inhibit the Notch signaling pathway, thus inhibiting related angiogenesis [63]. Regarding the Notch signaling pathway, it is important to explore the antiangiogenic effects of drugs, and further explorations in the clinic are needed.

## 7. Crosstalk

Multiple pathways commonly interact during angiogenesis in CRC. Current studies found that the Notch signaling pathway was associated with the Wnt and VEGF signaling pathways in CRC angiogenesis. Many studies have suggested that the Wnt/β-catenin/TCF signaling pathway positively regulated Jagged1 expression to activate Notch expression in CRC [162]. As an angiogenesis-related gene, Jagged1 has been shown to be closely associated with poor prognosis of CRC by regulating blood supply and tumor growth [163]. In detail, Notch1-mediated control of phosphorylated β-catenin could negatively regulate the Wnt/β-catenin signaling pathway [164]. Dll4, an important ligand of the Notch signaling pathway, is a downstream molecule of the VEGF pathway [145]. It promoted the transformation of normal blood vessels into tumor vessels by upregulating VEGFR3 and downregulating VEGFR1. This also suggested the importance of Dll4 and Notch pathway-targeted therapy in CRC angiogenesis. Tan IIA could regulate CRC cells via the cyclooxygenase-2-Wnt/β-catenin signaling pathway. Tan IIA downregulated the level of cyclooxygenase-2 and activated the Wnt/β-catenin pathway, which could inhibit CRC and lower the expression of VEGF [165]. In addition, β-catenin could also combine with TCF/LEF and activate VEGF [166].

Several studies have confirmed that NF-κB could interact with VEGF to jointly promote angiogenesis. These interactions included the aforementioned B7-H3/NF-κB/ VEGFA axis and the AKT/NF-κB/FOXD1/VEGFA pathway [9,69]. Furthermore, IκBα could be degraded by calpain-2 as a calcium-activated cysteine endopeptidase, thereby translocating NF-κB to the nucleus and inducing VEGF [167]. In addition, the activation of the AKT/NF-κB pathway could promote the secretion of VEGFA [70]. The EGFR/AKT/NF-κB pathway was involved in promoting angiogenesis by stimulating the production of VEGFA and IL-8 [168]. Furthermore, CCR6 was a CCR chemokine receptor. The AKT/NF-κB pathway played an important role in CCR6-mediated tumor angiogenesis. The Wnt/β-catenin signaling pathway was also correlated with other signaling pathways in CRC cancer, especially active β-catenin. Cyclin-dependent kinase 8 module (CDK8) regulated several relevant signaling pathways, including the Wnt/β-catenin signaling pathway. The CDK8 module and its analog CDK19 affected downstream transcription factors such as NF-κB and transcribed target genes [169]. CRC angiogenesis could also be affected by the regulation of NF-κB and β-catenin through the PI3K/AKT/IKKα pathway [49].

In addition to the above, some compounds could influence interactions in multiple pathways. Enalapril could significantly enhance the sensitivity of CRC to 5-FU and its antitumor effect by inhibiting NF-κB/STAT3 regulatory protein, proliferation and angiogenesis [170]. Crocin could remarkably inhibit CRC cells metastasis and angiogenesis by blocking the TNF-α/NF-κB/VEGF pathway [171]. Moreover, curcumin and its analogs significantly inhibited VEGFA synthesis and secretion in cell lines, suggesting that the inhibition of NF-κB was associated with p-STAT3 expression [172].

## 8. Conclusions

In this review, we summarized the pathways that influence angiogenesis in CRC. The specific content includes the basic introduction of the pathway, mechanism, some promoting and inhibiting factors of the pathway, targeted therapy and crosstalk. This review introduced not only the interactions among signaling pathways and some drugs already in clinical use but also the natural compounds that affect the crosstalk of multiple signaling pathways, which have great value in the research of targeted therapy for signaling pathways. We anticipated that these natural compounds could lead to new directions in the treatment of CRC in the future. Although many studies have focused on the role of signaling pathways in CRC angiogenesis, further research is needed to find more effective therapeutic agents.

## Figures and Tables

**Table 1:** Existing/potential drugs with anti-angiogenic effects in CRC.

Signaling Pathways	Drugs	Target	Classification	Functions	References
VEGF signaling pathway	Bevacizumab	VEGFA	Clinical	Monoclonal antibody for VEGFA	[36]
	Ramuciumab	VEGFR2	Clinical	Monoclonal antibody for VEGFR2	[39]
	Scopoletin	VEGFR2	Under study	VEGFR2 inhibitor	[41]
	Ginkgetin and Resveratrol	VEGFR2 and AKT	Under study	VEGFR2 and AKT inhibitor	[45]
	Tan IIA	HIF-1α and TGF-β1	Under study	HIF-1α inhibitor	[46]
	4‘-HW	PI3K and AKT	Under study	VEGFA inhibitor	[47]
NF-κB signaling pathway	Imatinib	VEGFR and ERK	Clinical	Tyrosine kinase inhibitor	[48]
	Curcumin	NF-κB	Clinical	NF-κB inhibitor	[49,50]
	Andrographolide	NF-κB	Under study	NF-κB inhibitor	[51]
	Parthenolide	NF-κB	Under study	NF-κB inhibitor	[52]
STAT3 signaling pathway	Aflibercept	IL-6	Clinical	STAT3 inhibitor	[53]
	Ponatinib	IL-6/1L-11/EGF	Clinical	STAT3 inhibitor	[54]
	Napabucasin	IL-6	Clinical	ROS and STAT3 inhibitor	[55]
	Convallatoxin	JAK2	Under study	STAT3 inhibitor	[56]
Wnt signaling pathway	Vitamin D	VDR	Clinical	Mxd1/Mad1 inducer and *c-Myc* inhibitor	[57,58,59]
	SAH-BCL9	β-catenin/BCL-9 complex	Under study	β-catenin and Wnt inhibitor	[60]
Notch signaling pathway	Berberine	Notch1	Clinical	Notch1 inhibitor	[61]
	ABL001	DLL4 and Notch1	Under study	DLL4/ NOTCH and VEGF/VEGFR inhibitor	[62]
	Quercetin	Notch1 and γ-secretase	Under study	Notch1 and γ-secretase inhibitor	[63]

## Data Availability

Not applicable.

## Abbreviations

AP	Andrographolide
BAFF	B cell-activating factor
BBR	berberine
BCL9	B-cell CLL/lymphoma 9
bFGF	basic fibroblast growth factor
BPA	Bisphenol A
BRG1	Brahma-related gene 1
CCL19	chemokine CC ligand 19
CELSR1	Cadherin EGF LAG seven-pass G-type receptors 1
CK1α	casein kinase 1α
CRC	colorectal cancer
CTD	Cantharidin
CXCR4	CXC chemokine receptor 4
DKK-1	Dickkopf-1
Dll1	Delta-like-1
Dll3	Delta-like-3
Dll4	Delta-Like-4
Dvl	disheveled
EERAC	Ethanol extracted from radix of Actinidia chinensis
EMT	epithelial to mesenchymal transition
eNOS	endothelial nitric oxide synthases
Gab2	Grb2-associated binder 2
HMGB1	High mobility group box 1
HUVECs	human umbilical vein endothelial cells
IKK	IκB kinase complex
IL-1	interleukin-1
ISM1	Isthmin 1
JAK	janus kinase
LRP5/6	low-density lipoprotein receptor-related protein 5/6
MAML1	mastermind like transcriptional coactivator 1
Mang-NPs	α-Mangostin-encapsulated PLGA nanoparticles
MAPK	mitogen-activated protein kinase
MCRC	metastatic colorectal cancer
MIP-3B	macrophage inflammatory protein 3-β
MTDH	metadherin
NF-κB	Nuclear Factor-kappa B
NF90	Nuclear Factor 90
NHERF2	Na^+^/H^+^ exchanger regulatory factor 2
NICD	Notch intracellular domain
NIK	NF-κB-inducing kinase
NLS	nuclear localization sequence
PLGF	placental growth factor
PRL-3	protein phosphatase of regenerating liver-3
PT	Parthenolide
Q8	(E)-2-(2-quinolin-2-yl-vinyl)-benzene-1, 4-diol HCl
RANKL	receptor-activated NF-κB ligand
ROS	reactive oxygen species
RXR	retinoid X receptor
SAFB	scaffold attachment factor B
SAH-BCL9	Stabilized Alpha-Helix of BCL9
SFRPs	Secreted frizzled-related proteins
SLC6A14	Solute carrier family 6 member 14
SRC	non-receptor protein tyrosine kinase
SRCIN1	SRC kinase signaling inhibitor 1
SRF	serum response factor
STAT	signal transducer and activator of transcription
Tan ⅡA	Tanshinone ⅡA
TAMs	Tumor associated macrophages
TGM2	Transglutaminase 2
TLR4	Toll-like receptor 4
TME	tumor microenvironment
TM4SF1	Transmembrane-4 L-six family member-1
TNF-α	tumor necrosis factor-α
VDR	vitamin D receptor
VDRE	vitamin D response element
VEGF	vascular endothelial growth factor
Wnt	Wingless and int-1
4’-HW	4’-hydroxywogonin
